# (5*E*)-5-(2,4-Dichloro­benzyl­idene)-2-(piperidin-1-yl)-1,3-thia­zol-4(5*H*)-one

**DOI:** 10.1107/S1600536811040785

**Published:** 2011-10-08

**Authors:** Hoong-Kun Fun, Madhukar Hemamalini, Prajwal L. Lobo, D. Jagadeesh Prasad, Boja Poojary

**Affiliations:** a X-ray Crystallography Unit, School of Physics, Universiti Sains Malaysia, 11800 USM, Penang, Malaysia; b Department of Chemistry, Mangalore University, Mangalagangothri 574 199, Mangalore, Karnataka, India

## Abstract

In the title compound, C_15_H_14_Cl_2_N_2_OS, the piperidine ring adopts a chair conformation. The dihedral angle between the thia­zolidine ring and the dichloro­benzene ring is 9.30 (4)°; this near coplanar conformation is stabilized by the formation of an intra­molecular C—H⋯S hydrogen bond, which generates an *S*(6) ring. In the crystal, mol­ecules are linked by C—H⋯O hydrogen bonds, forming [001] chains. Weak π–π inter­actions [centroid–centroid separation = 3.5460 (5) Å] consolidate the structure.

## Related literature

For details and properties of the 4-thia­zolidinone ring system, see: Lesyk & Zimenkovsky (2004 ▶); Lesyk *et al.* (2007 ▶); Havrylyuk *et al.* (2009 ▶); Ahn *et al.* (2006 ▶); Park *et al.* (2008 ▶); Geronikaki *et al.* (2008 ▶); Zimenkovsky *et al.* (2005 ▶). For ring puckering, see: Cremer & Pople (1975 ▶). For hydrogen-bond motifs, see: Bernstein *et al.* (1995 ▶). For the stability of the temperature controller used in the data collection, see: Cosier & Glazer (1986 ▶).
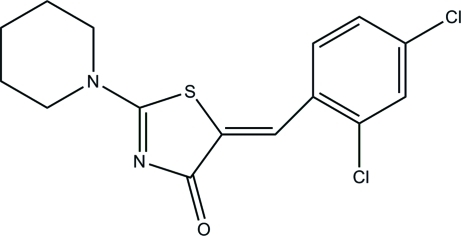


## Experimental

### 

#### Crystal data

C_15_H_14_Cl_2_N_2_OS
*M* *_r_* = 341.24
Monoclinic, 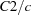

*a* = 28.5303 (3) Å
*b* = 7.4915 (1) Å
*c* = 15.4789 (2) Å
β = 116.407 (1)°
*V* = 2963.17 (6) Å^3^
*Z* = 8
Mo *K*α radiation
μ = 0.58 mm^−1^
*T* = 100 K
0.44 × 0.25 × 0.13 mm

#### Data collection

Bruker SMART APEXII CCD diffractometer
Absorption correction: multi-scan (*SADABS*; Bruker, 2009 ▶) *T*_min_ = 0.783, *T*_max_ = 0.928
46944 measured reflections
6673 independent reflections
5955 reflections with *I* > 2σ(*I*)
*R*_int_ = 0.024

#### Refinement

*R*[*F* ^2^ > 2σ(*F* ^2^)] = 0.026
*wR*(*F* ^2^) = 0.074
*S* = 1.03
6673 reflections
190 parameters
H-atom parameters constrained
Δρ_max_ = 0.52 e Å^−3^
Δρ_min_ = −0.19 e Å^−3^

### 

Data collection: *APEX2* (Bruker, 2009 ▶); cell refinement: *SAINT* (Bruker, 2009 ▶); data reduction: *SAINT*; program(s) used to solve structure: *SHELXTL* (Sheldrick, 2008 ▶); program(s) used to refine structure: *SHELXTL*; molecular graphics: *SHELXTL*; software used to prepare material for publication: *SHELXTL* and *PLATON* (Spek, 2009 ▶).

## Supplementary Material

Crystal structure: contains datablock(s) global, I. DOI: 10.1107/S1600536811040785/hb6435sup1.cif

Structure factors: contains datablock(s) I. DOI: 10.1107/S1600536811040785/hb6435Isup2.hkl

Supplementary material file. DOI: 10.1107/S1600536811040785/hb6435Isup3.cml

Additional supplementary materials:  crystallographic information; 3D view; checkCIF report

## Figures and Tables

**Table 1:** Hydrogen-bond geometry (Å, °)

*D*—H⋯*A*	*D*—H	H⋯*A*	*D*⋯*A*	*D*—H⋯*A*
C1—H1*A*⋯S1	0.95	2.49	3.2260 (8)	134
C4—H4*A*⋯O1^i^	0.95	2.40	3.3080 (9)	160
C15—H15*A*⋯O1^ii^	0.99	2.57	3.2778 (11)	129

Symmetry codes: (i) 
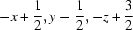
; (ii) 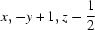.

**Fig. 1.**
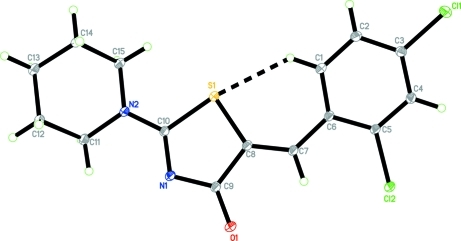
The asymmetric unit of the title compound, showing 50% probability displacement ellipsoids. An intramolecular hydrogen bond is shown by a dashed line.

**Fig. 2.**
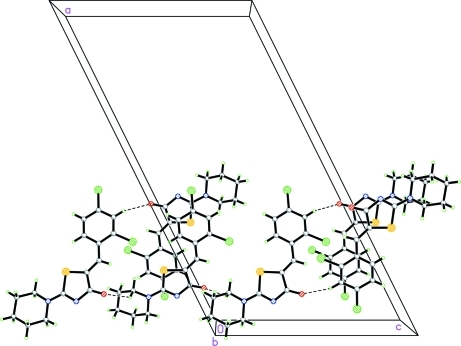
The crystal packing of the title compound (I).

## supplementary crystallographic information

### Comment

The 4-thiazolidinone ring system is a core structure in various synthetic compounds displaying a broad spectrum of biological activities (Lesyk & Zimenkovsky, 2004), including an anticancer effect (Lesyk *et al.*, 2007; Havrylyuk *et al.*, 2009). The mechanisms of antitumor activity by 4-thiazolidinones and related heterocycles may be associated with their affinities to anticancer bio-targets, such as phosphatase of a regenerating liver (PRL-3) (Ahn *et al.*, 2006; Park *et al.*, 2008) and nonmembrane protein tyrosine phosphatase (SHP-2)(Geronikaki *et al.*, 2008). 5-Arylidene derivatives were previously shown as the most active group of compounds with the anticancer activity among a large pool of 4-azolidone derivatives and analogs (Zimenkovsky *et al.*, 2005). This prompted us to synthesize the title compound, (I), (Fig. 1).

The piperidine ((N2/C11–C15) ring adopts a chair conformation [Q = 0.5462 (10) Å; θ = 5.78 (10)° and φ = 206.6 (10)°; Cremer & Pople, 1975].  The central thiazolidine (S1/N1/C8–C10) ring makes dihedral angles of 21.18 (4)° and 9.30 (4)°  with the terminal piperidine (N2/C11–C15) and phenyl (C1–C6) rings.  The corresponding angle between the piperidine and phenyl (N2/C11–C15)/(C1–C6) rings is 13.69 (4)°. An intramolecular C1—H1A···S1 hydrogen bond generates an *S*(6) (Bernstein *et al.*, 1995) ring motif.

In the crystal structure, (Fig. 2), the molecules are connected *via* intermolecular C—H···O (Table 1) hydrogen bonds forming one-dimensional supramolecular chains along the *c*-axis.  The crystal structure is further stabilized by weak π–π interactions between the thiazolidine (Cg1; S1/N1/C8–C10) and phenyl (Cg3; C1–C6) rings [Cg1···Cg3 = 3.5460 (5) Å; 1/2-x, 3/2-y, 1-z].

### Experimental

An equimolar mixture of 2-(4-methylsulfanylphenyl)acetohydrazide and 4-chlorobenzaldehyde was refluxed for four hours in the presence of few drops of acid catalyst and ethanol as solvent. The compound obtained was filtered, washed, dried and recrystalised from ethanol to yield brown blocks of (I).

### Refinement

All hydrogen atoms were positioned geometrically [ C–H = 0.95 or 0.99 Å] and were refined using a riding model, with *U*_iso_(H) = 1.2 *U*_eq_(C).

### Crystal data

**Table:**

C_15_H_14_Cl_2_N_2_OS	*F*(000) = 1408
*M**_r_* = 341.24	*D*_x_ = 1.530 Mg m^−^^3^
Monoclinic, *C*2/*c*	Mo *K*α radiation, λ = 0.71073 Å
Hall symbol: -C 2yc	Cell parameters from 9844 reflections
*a* = 28.5303 (3) Å	θ = 2.7–35.2°
*b* = 7.4915 (1) Å	µ = 0.58 mm^−^^1^
*c* = 15.4789 (2) Å	*T* = 100 K
β = 116.407 (1)°	Block, brown
*V* = 2963.17 (6) Å^3^	0.44 × 0.25 × 0.13 mm
*Z* = 8	

### Data collection

**Table:**

Bruker SMART APEXII CCD diffractometer	6673 independent reflections
Radiation source: fine-focus sealed tube	5955 reflections with *I* > 2σ(*I*)
graphite	*R*_int_ = 0.024
φ and ω scans	θ_max_ = 35.4°, θ_min_ = 2.7°
Absorption correction: multi-scan (*SADABS*; Bruker, 2009)	*h* = −45→46
*T*_min_ = 0.783, *T*_max_ = 0.928	*k* = −12→12
46944 measured reflections	*l* = −25→25

### Refinement

**Table:**

Refinement on *F*^2^	Primary atom site location: structure-invariant direct methods
Least-squares matrix: full	Secondary atom site location: difference Fourier map
*R*[*F*^2^ > 2σ(*F*^2^)] = 0.026	Hydrogen site location: inferred from neighbouring sites
*wR*(*F*^2^) = 0.074	H-atom parameters constrained
*S* = 1.03	*w* = 1/[σ^2^(*F*_o_^2^) + (0.0364*P*)^2^ + 1.7424*P*] where *P* = (*F*_o_^2^ + 2*F*_c_^2^)/3
6673 reflections	(Δ/σ)_max_ = 0.002
190 parameters	Δρ_max_ = 0.52 e Å^−^^3^
0 restraints	Δρ_min_ = −0.19 e Å^−^^3^

### Special details

**Table:**

Experimental. The crystal was placed in the cold stream of an Oxford Cryosystems Cobra open-flow nitrogen cryostat (Cosier & Glazer, 1986) operating at 100.0 (1) K.
Geometry. All s.u.'s (except the s.u. in the dihedral angle between two l.s. planes) are estimated using the full covariance matrix. The cell s.u.'s are taken into account individually in the estimation of s.u.'s in distances, angles and torsion angles; correlations between s.u.'s in cell parameters are only used when they are defined by crystal symmetry. An approximate (isotropic) treatment of cell s.u.'s is used for estimating s.u.'s involving l.s. planes.
Refinement. Refinement of F^2^ against ALL reflections. The weighted R-factor wR and goodness of fit S are based on F^2^, conventional R-factors R are based on F, with F set to zero for negative F^2^. The threshold expression of F^2^ > 2σ(F^2^) is used only for calculating R-factors(gt) etc. and is not relevant to the choice of reflections for refinement. R-factors based on F^2^ are statistically about twice as large as those based on F, and R- factors based on ALL data will be even larger.

### Fractional atomic coordinates and isotropic or equivalent isotropic displacement parameters (Å^2^)

**Table:**

	*x*	*y*	*z*	*U*_iso_*/*U*_eq_	
Cl1	0.430128 (8)	0.28966 (3)	0.729616 (15)	0.02400 (5)	
Cl2	0.269296 (8)	0.51920 (3)	0.790208 (13)	0.02382 (5)	
S1	0.176998 (7)	0.57324 (3)	0.374212 (12)	0.01596 (4)	
O1	0.11470 (2)	0.76041 (9)	0.52929 (4)	0.02178 (12)	
N1	0.09082 (3)	0.72148 (9)	0.36763 (5)	0.01686 (11)	
N2	0.08873 (3)	0.66216 (10)	0.21783 (4)	0.01825 (12)	
C1	0.28809 (3)	0.46653 (11)	0.54975 (5)	0.01796 (13)	
H1A	0.2688	0.4850	0.4822	0.022*	
C2	0.33866 (3)	0.40104 (11)	0.58547 (6)	0.01884 (13)	
H2A	0.3540	0.3774	0.5433	0.023*	
C3	0.36659 (3)	0.37051 (10)	0.68400 (6)	0.01726 (12)	
C4	0.34481 (3)	0.40355 (11)	0.74672 (5)	0.01740 (12)	
H4A	0.3639	0.3797	0.8138	0.021*	
C5	0.29438 (3)	0.47245 (10)	0.70876 (5)	0.01601 (12)	
C6	0.26411 (3)	0.50678 (10)	0.60961 (5)	0.01517 (12)	
C7	0.21245 (3)	0.58738 (10)	0.57455 (5)	0.01636 (12)	
H7A	0.2028	0.6221	0.6235	0.020*	
C8	0.17611 (3)	0.62056 (10)	0.48355 (5)	0.01523 (12)	
C9	0.12450 (3)	0.70822 (10)	0.46399 (5)	0.01629 (12)	
C10	0.11223 (3)	0.66071 (10)	0.31348 (5)	0.01553 (12)	
C11	0.03308 (3)	0.71217 (13)	0.16554 (6)	0.02211 (15)	
H11A	0.0227	0.7796	0.2093	0.027*	
H11B	0.0114	0.6027	0.1450	0.027*	
C12	0.02289 (3)	0.82567 (13)	0.07750 (6)	0.02274 (15)	
H12A	0.0392	0.9444	0.0988	0.027*	
H12B	−0.0153	0.8434	0.0394	0.027*	
C13	0.04478 (3)	0.73902 (13)	0.01359 (6)	0.02228 (15)	
H13A	0.0257	0.6267	−0.0141	0.027*	
H13B	0.0398	0.8202	−0.0403	0.027*	
C14	0.10279 (3)	0.69973 (12)	0.07298 (6)	0.01982 (14)	
H14A	0.1221	0.8133	0.0961	0.024*	
H14B	0.1165	0.6393	0.0320	0.024*	
C15	0.11176 (4)	0.58124 (12)	0.15908 (6)	0.02219 (15)	
H15A	0.0958	0.4627	0.1359	0.027*	
H15B	0.1498	0.5640	0.1991	0.027*	

### Atomic displacement parameters (Å^2^)

**Table:**

	*U*^11^	*U*^22^	*U*^33^	*U*^12^	*U*^13^	*U*^23^
Cl1	0.01844 (8)	0.02974 (10)	0.02435 (9)	0.00495 (7)	0.01001 (7)	0.00259 (7)
Cl2	0.01920 (9)	0.04116 (12)	0.01293 (7)	0.00453 (7)	0.00879 (6)	0.00332 (7)
S1	0.01586 (8)	0.02028 (8)	0.01193 (7)	0.00101 (6)	0.00634 (6)	−0.00006 (6)
O1	0.0210 (3)	0.0317 (3)	0.0141 (2)	0.0032 (2)	0.0092 (2)	−0.0011 (2)
N1	0.0159 (3)	0.0229 (3)	0.0124 (2)	0.0001 (2)	0.0069 (2)	0.0001 (2)
N2	0.0163 (3)	0.0272 (3)	0.0112 (2)	0.0023 (2)	0.0060 (2)	0.0005 (2)
C1	0.0199 (3)	0.0215 (3)	0.0134 (3)	0.0007 (3)	0.0083 (2)	0.0006 (2)
C2	0.0208 (3)	0.0212 (3)	0.0169 (3)	0.0014 (3)	0.0105 (3)	0.0005 (2)
C3	0.0165 (3)	0.0178 (3)	0.0180 (3)	0.0004 (2)	0.0081 (2)	0.0008 (2)
C4	0.0167 (3)	0.0204 (3)	0.0146 (3)	−0.0003 (2)	0.0064 (2)	0.0014 (2)
C5	0.0164 (3)	0.0203 (3)	0.0125 (3)	−0.0014 (2)	0.0075 (2)	0.0005 (2)
C6	0.0158 (3)	0.0175 (3)	0.0126 (3)	−0.0016 (2)	0.0066 (2)	0.0004 (2)
C7	0.0166 (3)	0.0201 (3)	0.0127 (3)	−0.0010 (2)	0.0068 (2)	0.0003 (2)
C8	0.0159 (3)	0.0178 (3)	0.0126 (3)	−0.0014 (2)	0.0068 (2)	−0.0002 (2)
C9	0.0164 (3)	0.0196 (3)	0.0133 (3)	−0.0010 (2)	0.0070 (2)	0.0001 (2)
C10	0.0150 (3)	0.0189 (3)	0.0125 (3)	−0.0008 (2)	0.0060 (2)	0.0003 (2)
C11	0.0155 (3)	0.0365 (4)	0.0139 (3)	0.0009 (3)	0.0061 (2)	0.0022 (3)
C12	0.0190 (3)	0.0339 (4)	0.0148 (3)	0.0050 (3)	0.0070 (3)	0.0033 (3)
C13	0.0230 (4)	0.0303 (4)	0.0133 (3)	0.0015 (3)	0.0078 (3)	0.0010 (3)
C14	0.0220 (3)	0.0245 (4)	0.0159 (3)	0.0008 (3)	0.0111 (3)	−0.0006 (3)
C15	0.0252 (4)	0.0291 (4)	0.0138 (3)	0.0072 (3)	0.0100 (3)	0.0015 (3)

### Geometric parameters (Å, °)

**Table:**

Cl1—C3	1.7357 (8)	C6—C7	1.4560 (11)
Cl2—C5	1.7397 (7)	C7—C8	1.3498 (10)
S1—C8	1.7402 (7)	C7—H7A	0.9500
S1—C10	1.7839 (8)	C8—C9	1.5148 (11)
O1—C9	1.2265 (9)	C11—C12	1.5207 (12)
N1—C10	1.3186 (10)	C11—H11A	0.9900
N1—C9	1.3722 (10)	C11—H11B	0.9900
N2—C10	1.3261 (9)	C12—C13	1.5296 (12)
N2—C15	1.4690 (10)	C12—H12A	0.9900
N2—C11	1.4743 (11)	C12—H12B	0.9900
C1—C2	1.3851 (11)	C13—C14	1.5223 (12)
C1—C6	1.4075 (10)	C13—H13A	0.9900
C1—H1A	0.9500	C13—H13B	0.9900
C2—C3	1.3900 (11)	C14—C15	1.5252 (12)
C2—H2A	0.9500	C14—H14A	0.9900
C3—C4	1.3880 (11)	C14—H14B	0.9900
C4—C5	1.3893 (11)	C15—H15A	0.9900
C4—H4A	0.9500	C15—H15B	0.9900
C5—C6	1.4102 (10)		
			
C8—S1—C10	88.74 (3)	N1—C10—S1	117.13 (5)
C10—N1—C9	111.56 (6)	N2—C10—S1	118.84 (6)
C10—N2—C15	122.99 (7)	N2—C11—C12	111.35 (7)
C10—N2—C11	120.09 (6)	N2—C11—H11A	109.4
C15—N2—C11	115.73 (6)	C12—C11—H11A	109.4
C2—C1—C6	122.55 (7)	N2—C11—H11B	109.4
C2—C1—H1A	118.7	C12—C11—H11B	109.4
C6—C1—H1A	118.7	H11A—C11—H11B	108.0
C1—C2—C3	118.91 (7)	C11—C12—C13	111.84 (7)
C1—C2—H2A	120.5	C11—C12—H12A	109.2
C3—C2—H2A	120.5	C13—C12—H12A	109.2
C4—C3—C2	121.44 (7)	C11—C12—H12B	109.2
C4—C3—Cl1	119.28 (6)	C13—C12—H12B	109.2
C2—C3—Cl1	119.29 (6)	H12A—C12—H12B	107.9
C3—C4—C5	118.17 (7)	C14—C13—C12	109.79 (6)
C3—C4—H4A	120.9	C14—C13—H13A	109.7
C5—C4—H4A	120.9	C12—C13—H13A	109.7
C4—C5—C6	123.08 (7)	C14—C13—H13B	109.7
C4—C5—Cl2	116.79 (5)	C12—C13—H13B	109.7
C6—C5—Cl2	120.12 (6)	H13A—C13—H13B	108.2
C1—C6—C5	115.83 (7)	C13—C14—C15	110.80 (7)
C1—C6—C7	123.46 (7)	C13—C14—H14A	109.5
C5—C6—C7	120.64 (7)	C15—C14—H14A	109.5
C8—C7—C6	130.21 (7)	C13—C14—H14B	109.5
C8—C7—H7A	114.9	C15—C14—H14B	109.5
C6—C7—H7A	114.9	H14A—C14—H14B	108.1
C7—C8—C9	121.03 (7)	N2—C15—C14	110.69 (7)
C7—C8—S1	129.85 (6)	N2—C15—H15A	109.5
C9—C8—S1	109.10 (5)	C14—C15—H15A	109.5
O1—C9—N1	124.51 (7)	N2—C15—H15B	109.5
O1—C9—C8	122.08 (7)	C14—C15—H15B	109.5
N1—C9—C8	113.41 (6)	H15A—C15—H15B	108.1
N1—C10—N2	124.03 (7)		
			
C6—C1—C2—C3	1.25 (12)	C7—C8—C9—O1	3.74 (12)
C1—C2—C3—C4	0.26 (12)	S1—C8—C9—O1	−177.51 (7)
C1—C2—C3—Cl1	−179.63 (6)	C7—C8—C9—N1	−175.99 (7)
C2—C3—C4—C5	−1.49 (12)	S1—C8—C9—N1	2.77 (8)
Cl1—C3—C4—C5	178.41 (6)	C9—N1—C10—N2	−178.19 (8)
C3—C4—C5—C6	1.30 (12)	C9—N1—C10—S1	1.36 (9)
C3—C4—C5—Cl2	−177.86 (6)	C15—N2—C10—N1	−175.15 (8)
C2—C1—C6—C5	−1.41 (12)	C11—N2—C10—N1	−8.17 (12)
C2—C1—C6—C7	175.53 (8)	C15—N2—C10—S1	5.31 (11)
C4—C5—C6—C1	0.10 (11)	C11—N2—C10—S1	172.29 (6)
Cl2—C5—C6—C1	179.24 (6)	C8—S1—C10—N1	0.25 (7)
C4—C5—C6—C7	−176.93 (7)	C8—S1—C10—N2	179.82 (7)
Cl2—C5—C6—C7	2.21 (10)	C10—N2—C11—C12	140.61 (8)
C1—C6—C7—C8	7.99 (13)	C15—N2—C11—C12	−51.50 (10)
C5—C6—C7—C8	−175.21 (8)	N2—C11—C12—C13	51.51 (10)
C6—C7—C8—C9	−179.51 (7)	C11—C12—C13—C14	−55.21 (10)
C6—C7—C8—S1	2.03 (13)	C12—C13—C14—C15	56.91 (10)
C10—S1—C8—C7	177.01 (8)	C10—N2—C15—C14	−139.08 (8)
C10—S1—C8—C9	−1.60 (5)	C11—N2—C15—C14	53.41 (10)
C10—N1—C9—O1	177.67 (8)	C13—C14—C15—N2	−55.37 (9)
C10—N1—C9—C8	−2.61 (9)		

### Hydrogen-bond geometry (Å, °)

**Table:**

*D*—H···*A*	*D*—H	H···*A*	*D*···*A*	*D*—H···*A*
C1—H1A···S1	0.95	2.49	3.2260 (8)	134
C4—H4A···O1^i^	0.95	2.40	3.3080 (9)	160
C15—H15A···O1^ii^	0.99	2.57	3.2778 (11)	129

Symmetry codes: (i) −*x*+1/2, *y*−1/2, −*z*+3/2; (ii) *x*, −*y*+1, *z*−1/2.
